# Early glial activation, synaptic changes and axonal pathology in the thalamocortical system of Niemann–Pick type C1 mice

**DOI:** 10.1016/j.nbd.2011.12.027

**Published:** 2012-03

**Authors:** Sarah N.R. Pressey, David A. Smith, Andrew M.S. Wong, Frances M. Platt, Jonathan D. Cooper

**Affiliations:** a Pediatric Storage Disorders Laboratory, Department of Neuroscience and Centre for the Cellular Basis of Behaviour, MRC Centre for Neurodegeneration Research, James Black Centre, Institute of Psychiatry, King's College London, 125 Coldharbour Lane, London, SE5 9NU, UK; b Department of Pharmacology, University of Oxford, Mansfield Road, Oxford, OX1 3QT, UK

**Keywords:** Au1, primary auditory cortex, CPu, Caudate putamen, GFAP, glial fibrillary acidic protein, LEnt, lateral entorhinal cortex, LGNd, dorsal lateral geniculate nucleus, LSDs, Lysosomal storage disorders, MGN, medial geniculate nucleus, M1, primary motor cortex, NPC, Niemann–Pick type C, Rt, Reticular nucleus, SNAP-25, Synaptosomal-associated protein of 25 kDa, SNr, substantia nigra reticularis, S1BF, somatosensory barrelfield cortex, VAMP2, Vesicle associated membrane protein 2, VPM/VPL, ventral posterior nucleus, V1, primary visual cortex, Niemann–Pick type C, Neuropathology, Astrocytes, Microglia, Synapse, Neurodegeneration, Thalamus, Cortex, Axonal spheroids

## Abstract

Niemann–Pick disease type C (NPC) is an inherited lysosomal storage disease characterised by accumulation of cholesterol and glycosphingolipids. NPC patients suffer a progressive neurodegenerative phenotype presenting with motor dysfunction, mental retardation and cognitive decline. To examine the onset and progression of neuropathological insults in NPC we have systematically examined the CNS of a mouse model of NPC1 (*Npc1*^*−/−*^ mice) at different stages of the disease course. This revealed a specific spatial and temporal pattern of neuropathology in *Npc1*^*−/−*^ mice, highlighting that sensory thalamic pathways are particularly vulnerable to loss of NPC1 resulting in neurodegeneration in *Npc1^−/−^* mice. Examination of markers of astrocytosis and microglial activation revealed a particularly pronounced reactive gliosis in the thalamus early in the disease, which subsequently also occurred in interconnected cortical laminae at later ages. Our examination of the precise staging of events demonstrate that the relationship between glia and neurons varies between brain regions in *Npc1^−/−^* mice, suggesting that the cues causing glial reactivity may differ between brain regions. In addition, aggregations of pre-synaptic markers are apparent in white matter tracts and the thalamus and are likely to be formed within axonal spheroids. Our data provide a new perspective, revealing a number of events that occur prior to and alongside neuron loss and highlighting that these occur in a pathway dependent manner.

## Highlights

► Staging of neuropathology defined in *Npc1*^*−/−*^ mouse model of Niemann–pick type C. ► The relationship between atrophy, neuron loss and glial activation are explored. ► Pathology was most pronounced in interconnecting sensory thalamocortical pathways. ► Glial activation precedes the onset of neuron loss. ► Pre-synaptic markers are rearranged and white matter is atrophied.

## Introduction

Niemann–Pick type C (NPC) is one of more than 50 inherited lysosomal storage disorders (LSDs), and like many of these disorders, is characterised by progressive neurological decline (Brady et al., 1966; Crocker and Farber, 1958). Neurological signs include ataxia, mental retardation, tremors, vertical supranuclear gaze palsy and dementia (Garver et al., 2007; Vanier, 2010). This disease can present neonatally, in childhood, adolescence or during adulthood resulting in a broad clinical spectrum of disease severity (Imrie et al., 2007; Tang et al., 2010; Vanier, 2010).

NPC has historically been thought of as a cholesterol storage disorder, as cholesterol accumulates in the liver and spleen, and is redistributed in the brain (Garver et al., 2007). However a plethora of lipids accumulate in NPC disease including neutral glycosphingolipids, gangliosides, sphingomyelin and sphingosine (Vanier, 1999). In the brain, which is the major site of NPC disease pathology, there is significant accumulation of gangliosides and sphingolipids (Siegel and Walkley, 1994; Vanier, 1999; Zervas et al., 2001). There is currently no consensus on what the functional role of the NPC disease pathway is and which metabolite is the central player in pathogenesis (Lloyd-Evans and Platt, 2010).

NPC displays an autosomal recessive mode of inheritance, with approximately 95% of cases caused by mutations in the *NPC1* gene (Carstea et al., 1997; Greer et al., 1998) and the remaining cases caused by mutations in *NPC2* (Krull et al., 1993; Naureckiene et al., 2000). The NPC2 protein is a soluble cholesterol binding protein (Garver and Heidenreich, 2002; Krull et al., 1993), and although NPC1 is thought to contribute to the transport of lipids the precise function of this protein remains incompletely understood (Lloyd-Evans and Platt, 2010; Sturley et al., 2004).

*Npc1*-null mutant mice (*Npc1*^*−/−*^) (Loftus et al., 1997) have proved a useful tool for examining the impact of *Npc1* deficiency, recapitulating many biochemical, pathological, neurological and behavioural features of human NPC disease. These include cholesterol and glycosphingolipid storage, loss of Purkinje cells of the cerebellum, axonal swelling, neuronal vacuoles and progressive motor impairments such as ataxia, tremor and loss of co-ordination (Baudry et al., 2003; Morris et al., 1982; Pentchev et al., 1984; Võikar et al., 2002). Following a progressive loss of body weight these mice die between 10 and 12 weeks of age (Pentchev et al., 1984).

Despite a thorough knowledge of the genetic basis of NPC disease, the underlying pathogenesis of this disease remains unclear and has turned out to be complex. Forming appropriate hypotheses on the mechanisms of neurodegeneration requires prior knowledge of the neuropathology that occurs as a result of loss of the NPC1 protein. Although the entire central nervous system (CNS) lacks the *NPC1* gene, it is becoming apparent that the effects upon the CNS are selective. While the Purkinje neurons of the cerebellum are known to be particularly vulnerable to neurodegeneration (Higashi et al., 1993; Pentchev et al., 1984), less is known about the effects of NPC disease on the rest of the brain. Therefore, to increase our understanding of how *Npc1* deficiency impacts upon the CNS we have systematically examined the onset and progression of neuropathological events including glial activation, atrophy and neuron loss in *Npc1*^*−/−*^ mice.

## Material and methods

### *Npc1* deficient mice

A spontaneous mutant mouse model of NPC on a BALB/C background was used in this study (*Npc1*^*−/−*^) (Pentchev et al., 1984) with wild-type littermates (*+/+*) used as controls. *Npc1*^*−/−*^ mice were maintained by heterozygous mating and the genotype of offspring was determined by polymerase chain reaction, as described previously (te Vruchte et al., 2004). Mice were bred and housed under standard non-sterile conditions at the University of Oxford. All animal procedures were conducted using protocols approved by the UK Animals (Scientific Procedures) Act (1986).

### Histological analysis

To analyze the onset and progression of CNS pathology in *Npc1* deficient mice, the brains of mutant mice and age-matched controls (n = 5) were examined at 3 time points during their life span representing presymptomatic (3 weeks), early symptomatic (6 weeks) and late symptomatic (9 weeks).

*Npc1*^*−/−*^ and control mice were perfused and tissue processed for histology according to established protocols (Bible et al., 2004; Kielar et al., 2007; Pontikis et al., 2004). Briefly, mice were deeply anaesthetised with sodium pentobarbitone (1 g/kg) and transcardially perfused with phosphate buffered saline (PBS) (Sigma-Aldrich, Poole, UK), followed by a freshly made and filtered solution of 4% Paraformaldehyde (PFA) in 0.1 M phosphate buffer, pH 7.4. Brains were removed and post-fixed in 4% PFA at 4 °C for 24 h, then bisected along the midline. Single hemispheres were cryoprotected in 30% sucrose, 0.5% sodium azide in 50 mM tris buffered saline (TBS), pH 7.6 prior to cutting frozen sections. 40 μm frozen coronal sections through the rostrocaudal extent of the cortical mantle were collected one per well in 96 well plates containing a cryoprotective solution (30% ethylene glycol (Sigma-Aldrich), 15% sucrose, 0.05% sodium azide in TBS) (Bible et al., 2004). All subsequent histological analyses were performed blind to genotype.

### Nissl staining

To provide direct visualisation of neuronal cytoarchitecture every sixth section through each brain was slide mounted and Nissl stained with cresyl violet (Bible et al., 2004; Kielar et al., 2007). Briefly, slides were incubated in 0.05% cresyl fast violet (Merck, Darmstadt, DE), 0.05% acetic acid in water for 30mins at 60 °C, rinsed in deionised water, then differentiated through an ascending series of alcohols before clearing in xylene and coverslipped with DPX (VWR, Poole, UK) (Bible et al., 2004; Kielar et al., 2007).

### Immunohistochemistry

A standard immunohistochemical protocol was used to examine the distribution of markers of interest (Bible et al., 2004; Pressey et al., 2010), using either 3,3′-diaminobenzidine tetrahydrochloride (DAB) or fluorescent probes for visualisation.

For visualisation with DAB, endogenous peroxidase activity was quenched in 1% hydrogen peroxidase (VWR) in TBS for 15 min. Sections were then rinsed in TBS and blocked in 15% normal serum (Vector, Northampton, UK) in TBS with 0.3% Triton-X (TBS-T, Sigma-Aldrich), before incubation in the appropriate primary antibody; polyclonal rabbit anti-GFAP (DakoCytomation 1:4000, Ely, UK), rat anti-mouse F4/80 (Serotec 1:100, Oxford, UK), rat anti-CD68 (Serotec 1:2000), mouse anti-synaptophysin (Cambridge Bioscience 1:200, Cambridge, UK), mouse anti-synaptobrevin (VAMP2) (Synaptic systems 1:2000, Goettingen, Germany), mouse anti-SNAP25 (BD Biosciences 1:1000, Oxford, UK) in 10% normal serum in TBS-T overnight at 4 °C. Sections were next rinsed in TBS and incubated with the appropriate biotinylated secondary antibodies; swine anti-rabbit (DakoCytomation 1:1000), rabbit anti-rat (Vector 1:200) and goat anti-mouse (Vector 1:1000) for 2 h at room temperature. Subsequently, sections were rinsed in TBS, followed by incubation in avidin-biotin-peroxidase complex (Vectastain Elite ABC kit, Vector, 1:1000) in TBS for 2 h and then rinsed in TBS. To visualise immunoreactivity, sections were incubated in 0.05% DAB (Sigma-Aldrich) containing 0.001% hydrogen peroxide in TBS for up to 25 min, depending on the antigen and then rinsed in ice-cold TBS. Finally, sections were mounted on gelatine-chrome-coated *Superfrost* microscope slides (VWR), air-dried overnight, cleared in xylene, and coverslipped with DPX (VWR).

Immunofluorescence staining was used to investigate the potential co-localization of the pre-synaptic marker synaptophysin with the GABAergic marker GAD 65/67. Sections were blocked in 15% normal serum in TBS-T, then probed with the appropriate primary antibodies: mouse anti-synaptophysin (Cambridge Bioscience 1:200), rabbit anti-GAD 65/67 (Millipore 1:1000, Watford, UK) in 10% normal serum in TBS-T overnight at 4 °C. After being rinsed in TBS and incubated with the appropriate fluorescent secondary Alexa-fluor antibodies (Molecular Probes 1:1000, Invitrogen, Carlsbad, CA) for 2 h at room temperature, sections were rinsed in TBS and mounted on gelatine-chrome-coated microscope slides and coverslipped with fluoromount-G (Southern Biotech, Birmingham, AL).

### Regional volume, cortical thickness and cell number estimation

Stereological estimates were obtained using *StereoInvestigator* software (Microbrightfield Inc, Williston, VT), with regions of interest defined by referring to the neuroanatomical landmarks described in Hof et al. (2000) and in Paxinos and Franklin (2001). Cavalieri estimates of regional volume (Kielar et al., 2007; Pressey et al., 2010) were obtained from the Nissl stained sections described above. A sampling grid was superimposed over the region of interest and the number of points covering the relevant area was assessed. The volume in μm^3^ was estimated from a series of sections through each region of interest. Sampling grids and series used were as follows: Cortex, 1:12, 250 μm^2^; striatum, 1:12, 150 μm^2^; thalamus, 1:12, 200 μm^2^; hippocampus, 1:6, 200 μm^2^; corpus callosum, 1:12, 100 μm^2^ and internal capsule and cerebral peduncle, 1:6, 100 μm^2^. Analyses were carried out on an Olympus BX50 microscope (Olympus, Southend-on-Sea, UK) linked to a DAGE-MTI CCD-100 camera (Dage-MTI, Michigan City, IA).

To determine the regional distribution of cortical atrophy we measured the thickness of selected cortical sub-divisions; somatosensory barrelfield cortex (S1BF), primary visual cortex (V1), primary motor cortex (M1), primary auditory cortex (Au1) and lateral entorhinal cortex (LEnt). In each region ten perpendicular lines were traced from the pial surface of the cortex to the white matter of the corpus callosum and the mean length used as a measure of thickness. This was calculated in three consecutive sections starting from a defined rostrocaudal level for each region (Bible et al., 2004; Kielar et al., 2007).

The optical fractionator probe was used to estimate cell number (West et al., 1991) in Nissl stained sections of thalamic relay nuclei and lamina IV and VI of the S1BF (Kielar et al., 2007; Pressey et al., 2010). Nissl stained cells were counted, using a 100 × objective and counted as neurons if they had a neuronal morphology and a clearly identifiable nucleus. As such, astrocytes and microglia with their small soma, or cells with indistinct morphology were not counted. A line was traced around the boundary of the region of interest, a grid was superimposed and cells were counted within a series of dissector frames placed according to the sampling grid size. Different grid and dissector sizes were used in each brain region using a coefficient of error (CE) value of less than 0.1 to indicate sampling efficiency (Gundersen et al., 1999). For thalamic nuclei, a 1:6 series was sampled using the following sampling schemes: LGNd grid 125 × 125 μm, frame 74 × 42 μm; VPM, grid 175 × 175 μm, frame 74 × 42 μm; VPL, grid 100 × 100 μm, frame 74 × 42 μm. For the S1BF, a 1:12 series was sampled using the following sampling schemes: lamina IV, grid 150 × 150 μm, frame 41 × 26 μm; lamina VI, grid 200 × 200 μm, frame 60 × 40 μm.

### Western blotting

To determine the levels of synaptic protein expression, western blots were performed on crude synaptic preparations (Hruska et al., 1978; Kadota and Kadota, 1973). After cervical dislocation, brains were bisected along the midline and the left hemisphere was dissected to produce cortical and subcortical samples. Tissues were collected into ice cold homogenization buffer containing 4 mM HEPES (4-(2-hydroxyethyl)-1-piperazineethanesulfonic acid), 0.32 M sucrose, 1 mM MgCl_2_, 0.5 mM CaCl_2,_ pH7.3, containing proteinase inhibitors (ROCHE, Welwyn Garden City, UK) and homogenized with a Teflon homogenizer at 700 rpm with 15 up and down strokes. Samples were centrifuged at 1000 g for 10 min at 4 °C, the pellet was resuspended in 0.5 ml homogenization buffer and centrifuged again under the same conditions. Next, the combined supernatant was centrifuged at 13800 g for 15 min at 4 °C and the pellet was resuspended in 50 μl homogenization buffer (enriched synaptic fraction). The protein concentration was determined using a bicinchoninic acid protein kit (Thermo-Fischer Scientific, Northumberland, UK) and 2 μg from each sample was subjected to electrophoresis in a precast 10–20% Tris–HCl Mini-PROTEAN Gel (Bio-Rad, Hemel Hempstead, UK). Gels were blotted onto PVDF membranes, blocked with Odyssey blocking buffer (Li-COR Biosciences, Lincoln, NE) 1:1 PBS-Tween-20 (referred to as blocking buffer) for 1 h at room temperature and then incubated with the following primary antibodies diluted in blocking buffer at 4 °C overnight: mouse anti-synaptophysin (Cambridge Bioscience 1:1000), mouse anti-synaptobrevin (VAMP2) (Synaptic systems 1:10 000). After washing, membranes were incubated with fluorescent goat anti mouse IR-Dye 800 secondary antibody (Li-COR Biosciences 1:10 000) for 1 h at room temperature. After washing, immunoreactive bands were visualised using an Odyssey Infrared Imaging System (Li-COR Biosciences), at a scan resolution of 169 μm. Membranes were next stripped of antibodies using Restore-PLUS western blot stripping buffer (Thermo-Fischer Scientific) and reprobed for Mouse anti-Actin (Abcam, Cambridge UK 1:5000) as a loading control. Quantification was performed on single channels with the analysis software provided. Arbitrary fluorescence values were assigned to bands, giving relative fluorescence intensity between bands on each membrane and values were normalised to actin. Three mice were used for each brain region.

### Statistical analysis

Microsoft Excel (Redmond, WA) was used for data collection and SPSS (SPSS Inc. Chicago, IL) for statistical analysis. To test for significance between genotypes, the Student's *t*-test or ANOVA test with post hoc Bonferroni analysis were used as appropriate. All graphs are plotted as the mean ± the standard error of the mean (SEM).

## Results

### Regionally localised neuropathology in *Npc1*^*−/−*^ mice

The severe neurological phenotypes displayed by *Npc1*^*−/−*^ mice are accompanied by marked changes in both neurons and glia (Baudry et al., 2003; German et al., 2002; Suzuki et al., 2003), but the onset and progression of these events have not been characterised in detail. To further understand the time course of neuropathological changes in *Npc1*^*−/−*^ mice we have systematically examined the forebrain of *Npc1*^*−/−*^ mice at different stages of disease progression.

**Fig. 1:**
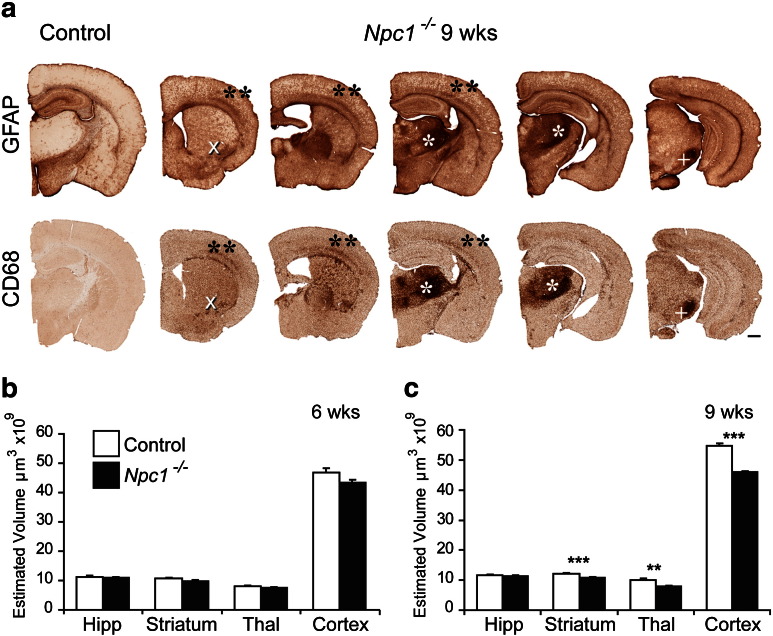
Prominent thalamic glial activation and widespread CNS atrophy in *Npc1^−/−^* mice. a Immunohistochemical staining for the astrocyte marker glial fibrillary acidic protein (GFAP) and the microglial marker CD68 revealed reactive glia throughout the CNS of *Npc1^−/−^* mice at disease end stage (9 weeks of age). Reactive astrocytes and microglia displayed a similar spatial distribution with the most intense immunoreactivity for both markers in the thalamus (*). In addition, localised gliosis was evident in lamina VI of primary somatosensory cortex (**), the ventral lateral striatum (X) and the substantia nigra (+) (Scale bar; 500 μm). b–c Cavalieri estimates of regional volume revealed significant atrophy of the striatum, thalamus and cortex, but not the hippocampus in 9 week–old *Npc1^−/−^* mice compared to wild type controls (c). This atrophy was only apparent at disease end stage, and was not present in 6 week-old *Npc1^−/−^* mice (b). (Hipp, Hippocampus; Thal, Thalamus; Error bars show SEM, n = 4–5, ** p < 0.01; *** p < 0.001).

Astrocytosis and microglial activation can be sensitive markers of ongoing neuronal dysfunction or injury (Raivich et al., 1999). To explore which regions of the brain suffer injury in *Npc1*^*−/−*^ mice, we used the astrocyte marker GFAP (Eng et al., 2000) and the microglial marker CD68 (Kunisch et al., 2004), initially to perform a low power survey of severely affected *Npc1*^*−/−*^ mice (9 weeks). This revealed widespread activation of both astrocytes and microglia and highlighted the thalamus as a focus of this immune reaction compared to other brain regions (Fig. 1a*). Prominent staining for both GFAP and CD68 was also present in the deeper laminae VI of primary sensory cortical regions (Fig. 1a**), the substantia nigra (Fig. 1a +) and to a lesser extent in the ventral lateral striatum (Fig. 1a X). In comparison, activation of both astrocytes and microglia was more diffuse in the hippocampus of *Npc1*^*−/−*^ mice (Fig. 1a), with no sub-field displaying more activation than any other. At this magnification the overall atrophy of the 9 week-old *Npc1*^*−/−*^ brain was also apparent. Using the Cavalieri stereological probe for regional volume we found that this was a result of significantly reduced volume of the striatum, thalamus and cortex, with only the hippocampus remaining unaffected in *Npc1*^*−/−*^ mice at 9 weeks of age (Fig. 1c). Examination of younger mice revealed that this atrophy appeared to be a relatively late stage phenotype, with no atrophy of these regions evident in 6 week-old mutant mice (Fig. 1b).

### Localised thalamic glial activation

**Fig. 2:**
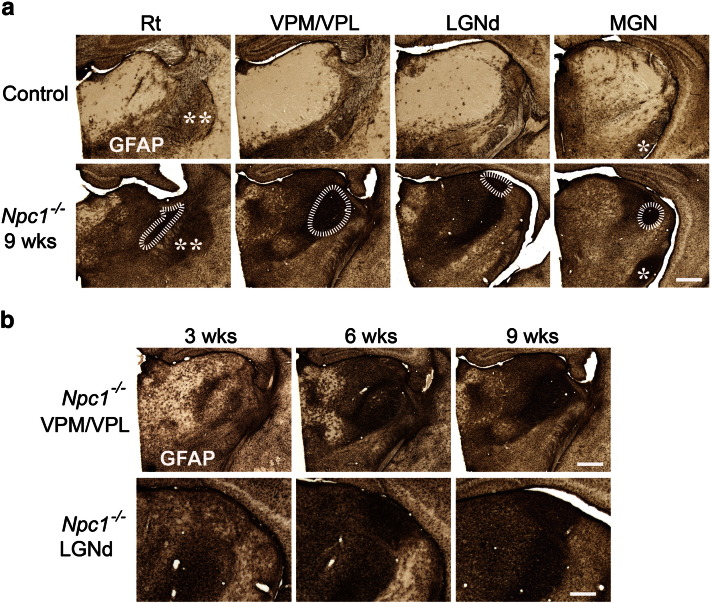
Pronounced astrocytosis in the thalamus of *Npc1^−/−^* mice. Immunohistochemical staining for glial fibrillary acidic protein (GFAP) revealed striking upregulation of this marker in the thalamus of *Npc1^−/−^* mice compared to control mice (+/+). a GFAP immunoreactivity was most intense in the reticular thalamic nucleus (Rt), the ventral posterior nucleus (VPM/VPL), the dorsal lateral geniculate nucleus (LGNd) and the medial geniculate nucleus (MGN) in 9 week-old *Npc1^−/−^* mice (The intense GFAP immunoreactivity adjacent to the LGNd is a more posterior portion of the VPM/VPL). b Reactive astrocytosis was already evident in the VPM/VPL and LGNd of 3 week-old *Npc1^−/−^* mice and progressed in intensity with age. Basal ganglia nuclei indicated by; ** Globus pallidus, * Substantia nigra (Scale bar = 500 μm, except in B LGNd pictures where scale bar = 250 μm).

**Fig. 3:**
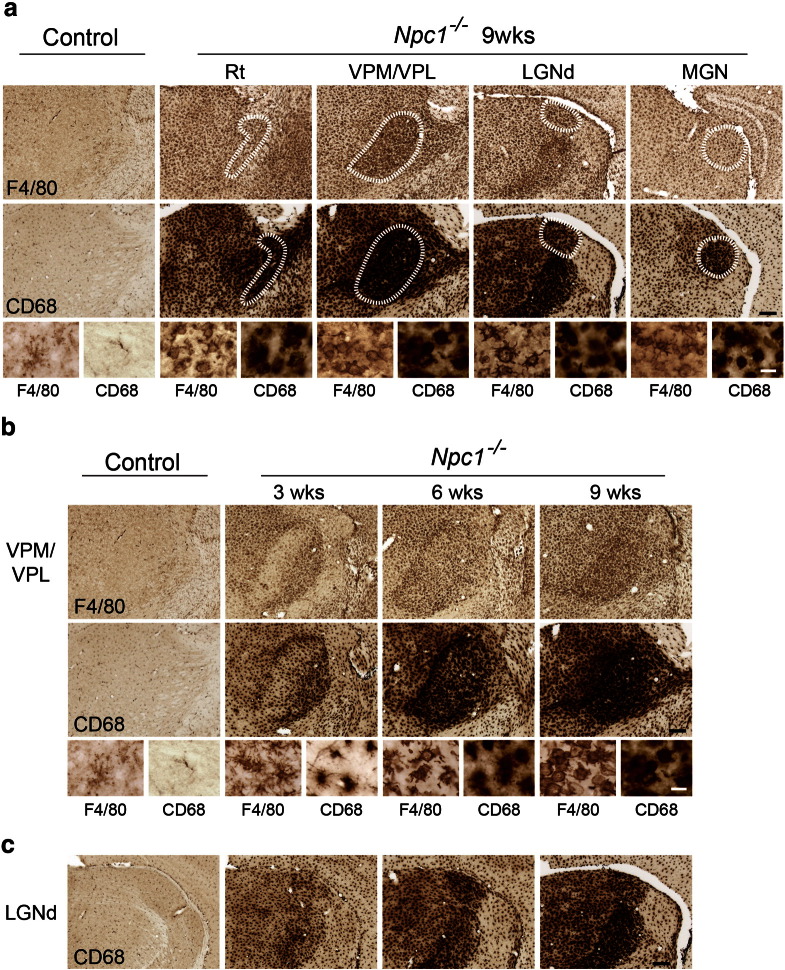
Profound microglial activation in the thalamus of *Npc1^−/−^* mice. Immunohistochemical staining for the microglial markers F4/80 and CD68 revealed upregulation of these markers in the thalamus of *Npc1*^*−/−*^ mice compared to controls. a F4/80 and CD68 immunoreactivity was most striking in the reticular thalamic nucleus (Rt), the ventral posterior nucleus (VPM/VPL), the dorsal lateral geniculate nucleus (LGNd) and the medial geniculate nucleus (MGN) in 9 week-old *Npc1*^*−/−*^ mice. Higher power pictures reveal the amoeboid morphology of microglia indicating their activated state (The intense F4/80 and CD68 immunoreactivity in the LGNd panel is a more posterior portion of the VPM/VPL). b–c F4/80 and CD68 immunoreactivity was already evident in 3 week-old *Npc1*^*−/−*^ mice in selected thalamic nuclei including VPM/VPL (b) and LGNd (c). Higher power pictures (b) reveal the morphological transformation of microglia from a more ramified morphology at 3 weeks of age to amoeboid microglia by 6 weeks of age. (Scale bar: 200 μm in low power images; 20 μm in inserts).

Closer examination of GFAP (astrocytes), F4/80 and CD68 (microglia) immunoreactivity in the thalamus revealed that this intense reactive gliosis was made up of a series of selective effects within different thalamic nuclei during disease progression. Upregulation of these glial markers began early in pathogenesis, with localised patches of astrocytosis and reactive microglia already evident in 3 week-old *Npc1*^*−/−*^ mice. This gliosis was evident only in selected thalamic nuclei, the majority of which relay sensory information (Table 1, Figs. 2b, 3b–c). With disease progression, this reactive gliosis spread to encompass increasingly more thalamic nuclei (Table 1). The sensory nuclei that displayed the earliest reactive gliosis at 3 weeks of age (ventral posterior nucleus (VPM/VPL; somatosensory), the dorsal lateral geniculate nucleus (LGNd; visual) and the medial geniculate (MGN; auditory)), were those that later displayed the most pronounced gliosis in end-stage 9 week old *Npc1*^*−/−*^ mice (Figs. 2a, 3a). The progressive nature of this immune response was also evident through the morphological transformation of microglia. In 3 week-old *Npc1*^*−/−*^ mice F4/80 immunoreactivity revealed ramified microglia displaying multiple long processes with many branches (Fig. 3b, F4/80 inserts), indicating a low level of activation. However, by 6 weeks of age, microglia in these nuclei displayed amoeboid morphology with large round cell bodies and short thickened processes (Fig. 3a–b, inserts), an indication of activated brain macrophage-like state (Raivich et al., 1999).

### Localised cortical pathology

**Fig. 4:**
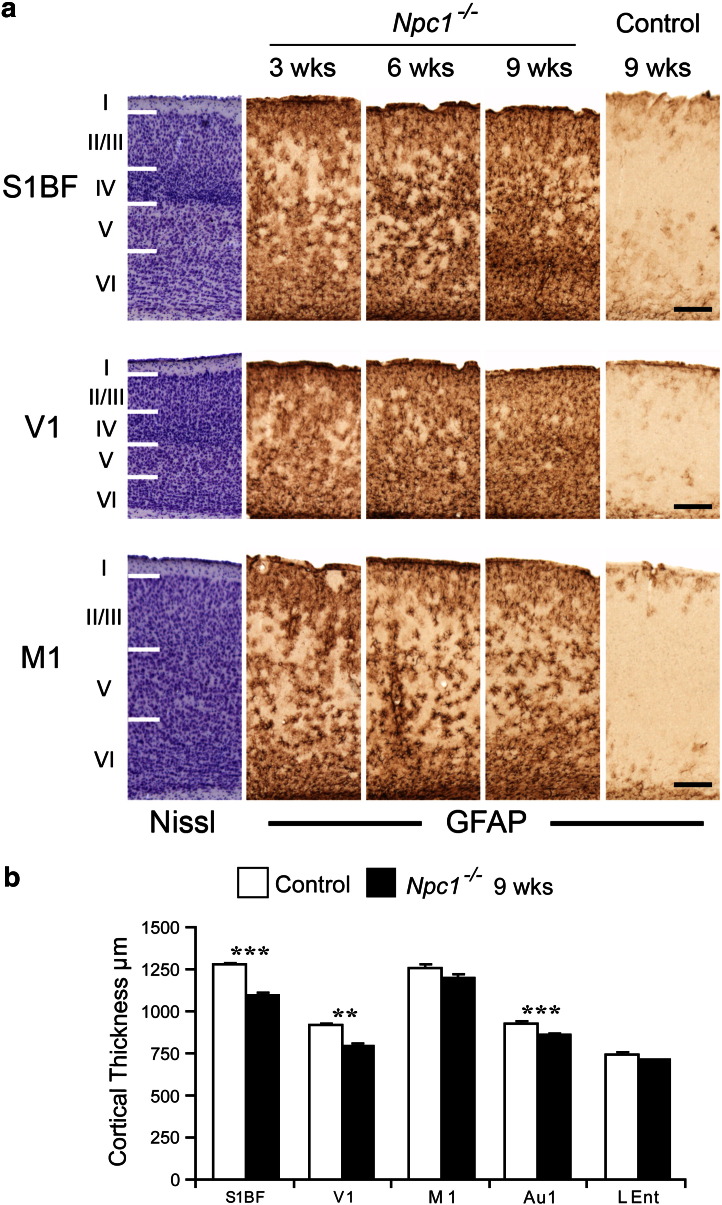
Cortical astrocytosis and atrophy in *Npc1^−/−^* mice. a Immunohistochemical staining for glial fibrillary acidic protein (GFAP) reveals astrocytosis in the somatosensory barrelfield cortex (S1BF), the primary visual cortex (V1) and the primary motor cortex (M1) in *Npc1*^*−/−*^ mice. Adjacent Nissl stained sections illustrate cortical laminar boundaries. Astrocytosis was already evident in all cortical subfields in 3 week old *Npc1*^*−/−*^ mice and increased in intensity with disease progression, with the greatest density of astrocytes in sensory regions (V1 and S1BF) compared to motor (M1). Bands of particularly dense astrocytosis became evident in lamina VI of the S1BF in 9 week-old *Npc1*^*−/−*^ mice (Scale bar; 200 μm). b Measurement of the thickness of cortical subfields revealed selective atrophy of sensory cortical regions S1BF, V1 and primary auditory cortex (Au1) in 9 week-old *Npc1*^*−/−*^ mice, but relative sparing of M1 and the lateral entorhinal cortex (LEnt). (Error bars show SEM, n = 4–5, **, p < 0.01, ***, p < 0.001).

**Fig. 5:**
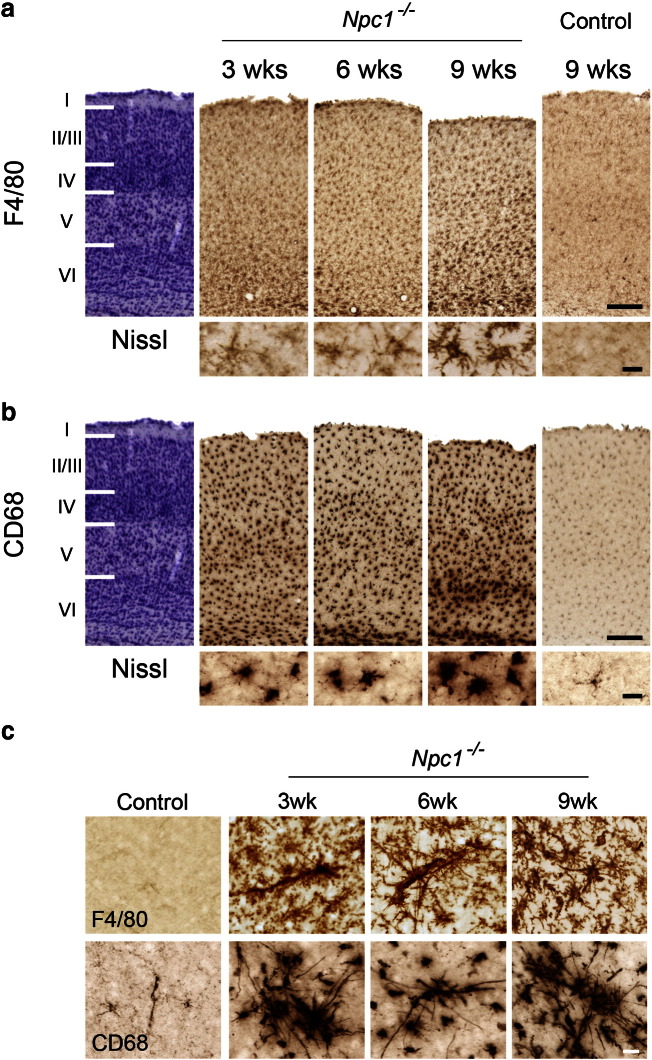
Microglial activation in the cortex of *Npc1^−/−^* mice. a, b Immunohistochemical staining for the F4/80 (a) and CD68 (b) demonstrate early microglial activation in the somatosensory barrelfield cortex (S1BF) of *Npc1*^*−/−*^ mice. Reactive microglia were evenly distributed throughout all laminae, displaying very little laminar specificity (adjacent Nissl stained sections illustrate cortical laminar boundaries). At the end stage of disease progression, a band of particularly dense microglia became evident in the dorsal part of lamina VI of the S1BF (a, b). c F4/80 and CD68 immunoreactivity reveal distinctive rod-like morphology of microglia in the cortex of *Npc1*^*−/−*^ mice. (Scale bar = 200 μm in low power cortical views in a and b, 20 μm in high power inserts and c).

In comparison to the thalamus, the cortex of *Npc1*^*−/−*^ mice displayed a much lower level of reactive gliosis (Fig. 1). However, similar to the events in the thalamus glial activation was most evident in sensory cortical regions. The most intense reactive gliosis seen in the cortex of *Npc1*^*−/−*^ mice was a localised band of densely packed astrocytes and microglia in the most dorsal part of lamina VI of the somatosenory barrelfield cortex (S1BF) (Figs. 4a, 5a–b). In addition, comparison of cortical subfields that serve different functions revealed that reactive astrocytes were more densely packed in the sensory S1BF and the primary visual cortex (V1), compared to the primary motor cortex (M1) (Fig. 4a). To examine whether the cortical atrophy evident in 9 week-old *Npc1*^*−/−*^ mice (Fig. 1c) was also selective for these different subfields we made thickness measurements of cortical subfields that serve different functions. This revealed selective atrophy of sensory regions of the cortex, whereas the lateral entorhinal (LEnt) and M1 were spared (Fig. 4b).

In addition, F4/80 immunoreactivity revealed that microglia were rarely morphologically transformed into brain macrophages in the cortex, with these cells maintaining long, branched processes, even at disease end stage (Fig. 5a inserts). However, in addition to these typical ramified microglia in the cortex of *Npc1*^*−/−*^ mice, there were occasional microglia with multiple long, straight branches extending much further than the usual reach of a single microglial cell (Fig. 5c). These rod-shaped F4/80 and CD68 positive cells were apparent in small clumps and sparsely distributed in the cortex, and were evident most often in sensory and ventral cortical regions adjacent to the corpus callosum.

### Staged neuron loss in the thalamocortical system

To examine the relationship between glial activation and neuron loss through the thalamocortical system we focussed upon the thalamic nuclei (VPM, VPL, LGNd) and cortical regions (S1BF), which displayed the most intense localised glial activation. This approach also enabled the examination of interconnected regions since the S1BF is the cortical target region of the VPM/VPL, where lamina IV granule neurons receive afferent inputs from the VPM/VPL and lamina VI neurons send efferent feedback projections to the thalamus.

**Fig. 6:**
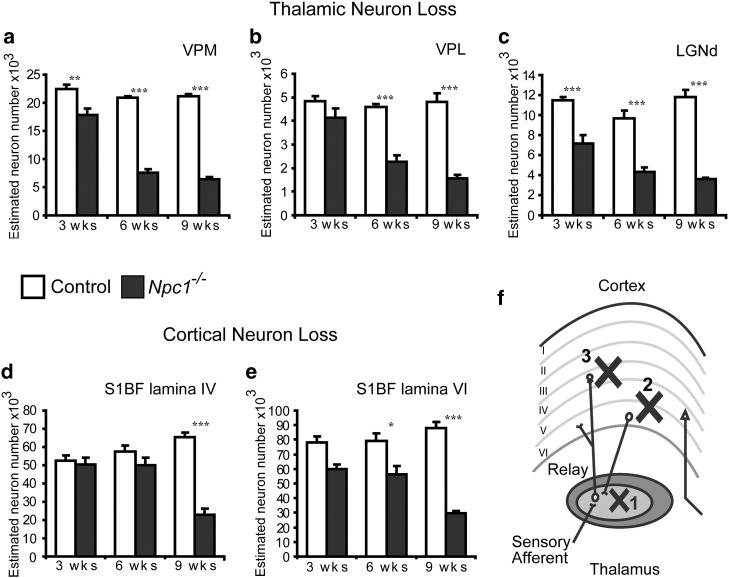
Sequential neuron loss in the thalamocortical system of *Npc1^−/−^* mice. **a**–**c** Optical fractionator estimates of neuron number in individual thalamic relay nuclei show significantly fewer neurons in the dorsal lateral geniculate nucleus (LGNd) and the ventral posterior medial nucleus (VPM) of *Npc1*^*−/−*^ mice from 3 weeks of age, compared to control mice and from 6 weeks of age in the ventral posterior lateral nucleus (VPL). **d**, **e** In the cortex of *Npc1*^*−/−*^ mice, optical fractionator estimates of neuron number reveal progressive loss of neurons in lamina VI of the S1BF, becoming significant at 6 weeks of age (**e**) compared to relatively delayed onset of neuron loss in lamina IV, which only became significant at 9 weeks of age (**d**). f Diagram illustrating the interconnected pathways between a thalamic relay nucleus and the cortex. Crosses indicate the order of neuron loss observed in the somatosensory relay system in *Npc1*^*−/−*^ mice. (Error bars show SEM; n = 4–5; * p < 0.05; ** p < 0.01; *** p < 0.001 using one way ANOVA).

Optical fractionator estimates of neuron number revealed that neuron loss was already evident in the LGNd and VPM thalamic nuclei in 3 week-old mutant mice (Figs. 6a, c). Only subsequently did neuron loss become significant in the VPL at 6 weeks of age (Fig. 6b). In all three thalamic nuclei there was a significant drop in neuron number in *Npc1*^*−/−*^ mice between 3 and 6 weeks of age (p < 0.05 ANOVA), suggesting that a substantial insult to neuron survival in these thalamic nuclei occurs between these ages. In contrast, neuron loss in S1BF was a later event, with a significant difference between *Npc1*^*−/−*^ mice and controls at 6 weeks of age in lamina VI (Fig. 6e), and at 9 weeks of age in lamina IV (Fig. 6d). In both these laminae, the most significant decrease in the number of neurons occurred between 6 and 9 weeks of age (p < 0.003, ANOVA), revealing a later onset neuron loss in the cortex compared to the thalamus of *Npc1*^*−/−*^ mice. Taken together, our data show localised pathological changes within interconnected thalamocortical pathways, with neuron loss apparent earlier in disease progression in the thalamus than the cortex, reflecting the pattern of reactive gliosis.

### Basal ganglia pathology

**Fig. 7:**
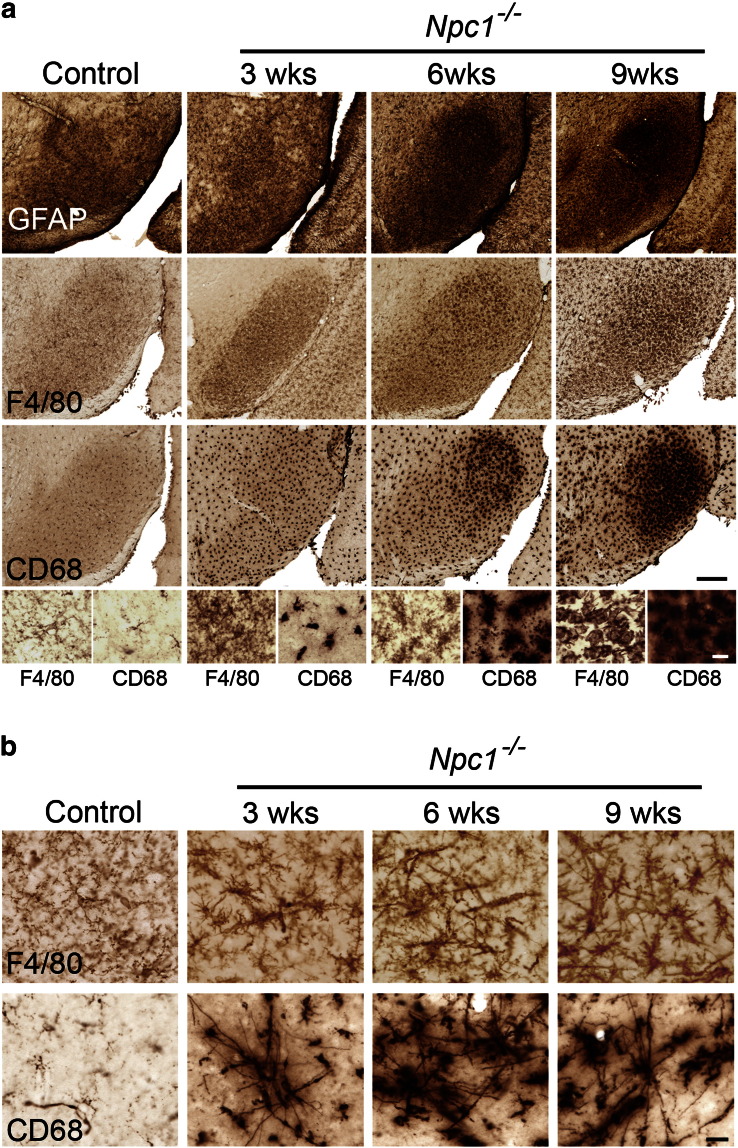
Localised glial activation in the substantia nigra and striatum of *Npc1^−/−^* mice. **a** GFAP immunoreactivity reveals an increased astrocytosis in the substantia nigra of *Npc1*^*−/−*^ mice from 6 weeks of age, with predominance for the dorsal part of the substantia nigra pars reticularis (SNr). F4/80 and CD68 immunoreactivity reveal that microglial activation precedes astrocytosis with microglia already activated from 3 weeks of age in the SNr. This microglial activation is progressive with an increased CD68 immunoreactivity in the dorsal part of the substantia nigra from 6 weeks, but with microglia not fully transformed into amoeboid microglia until 9 weeks of age (high power pictures). **b** F4/80 and CD68 immunoreactivity revealed microglia in the dorsal lateral part of the striatum to have an unusual rod-like morphology, which was already evident in 3 week old mice. (Scale bar in a = 200 μm and 10 μm in inserts; in b = 20 μm).

In addition to this thalamocortical pathology, the basal ganglia also displayed localised intense reactive gliosis (Fig. 1a). In the substantia nigra, a progressive upregulation of glial markers was evident from an early age, with partially transformed microglia evident in both the pars compacta and reticularis from 3 weeks of age (Fig. 7a). Subsequently, GFAP immunoreactivity was also increased in mutant mice at 6 weeks of age, and was more prominent in the dorsal part of the substantia nigra reticularis (SNr). This specificity for the dorsal part of the SNr was mirrored by CD68 immunoreactivity, but surprisingly, F4/80 immunoreactivity was distributed evenly throughout the SNr (Fig. 7a). Compared to this early onset reactive gliosis, pronounced gliosis in the globus pallidus was only evident at a late stage in the 9 week-old *Npc1*^*−/−*^ mice (Fig. 2a**).

In the striatum of *Npc1*^*−/−*^ mice, staining for either F4/80 or CD68 revealed many microglia with rod-like morphology (Fig. 7b). In contrast to the cortex (where rod-like microglia were sparsely distributed), multiple clumps of these cells were evident consistently in the ventral lateral part of the caudate putamen (CPu) of the striatum. This phenotype was an early event, with many rod-like microglia evident in 3 week-old *Npc1*^*−/−*^ mice. GFAP staining revealed that astrocytes were also upregulated in this region, but these astrocytes did not display any unusual morphological features and were only mildly hypertrophied (data not shown).

### Early white matter pathology

**Fig. 8:**
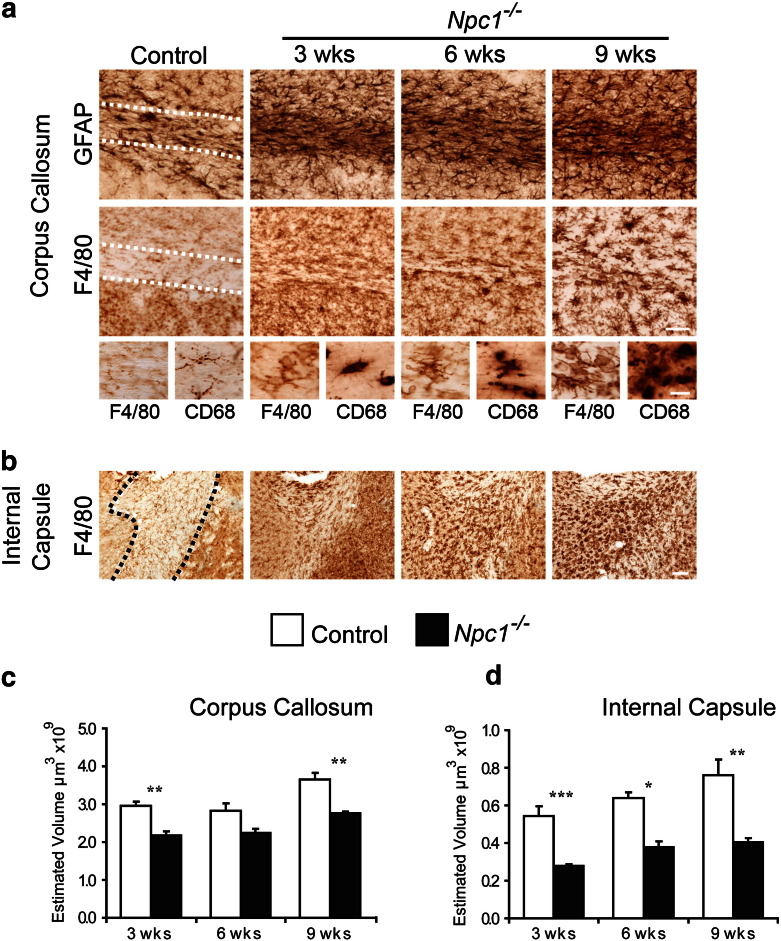
Reactive gliosis and atrophy of white matter in *Npc1^−/−^* mice. **a** Localised intense GFAP and F4/80 immunoreactivity were evident in the corpus callosum of *Npc1*^*−/−*^ mice. GFAP immunoreactivity revealed a high level of astrocytosis was already evident at 3 weeks of age and F4/80 immunoreactivity revealed microglia to have transformed into amoeboid morphology at this early age (inserts). **b** Likewise, very intense F4/80 immunoreactivity was evident in the internal capsule of *Npc1*^*−/−*^ mice from 3 weeks of age. **c–d** Cavalieri estimates reveal reduced volume of the corpus callosum and internal capsule throughout the life span of *Npc1*^*−/−*^ mice compared to age matched controls. (Scale bar in **b** = 50 μm and 20 μm in inserts; in **c** = 100 μm; Error bars show SEM; n = 4–5, * p < 0.05; ** p < 0.01; *** p < 0.001 using one way ANOVA).

Our survey of the *Npc1*^*−/−*^ forebrain also revealed that white matter tracts displayed particularly pronounced glial activation. In 3 week-old *Npc1*^*−/−*^ mice, many intensely stained astrocytes and microglia were apparent in the corpus callosum (Fig. 8a), internal capsule (Fig. 8b) and the cerebral peduncle (data not shown). Distinct from grey matter structures, in which microglia remained ramified in 3 week-old *Npc1*^*−/−*^ mice, we observed fully transformed amoeboid microglia in the white matter of 3 week-old *Npc1*^*−/−*^ mice (Fig. 8a inserts). This gliosis was associated with a consistently reduced volume of white matter tracts compared to controls at all stages of disease progression (Figs. 8c–d).

### Aberrant synaptic protein aggregation

**Fig. 9:**
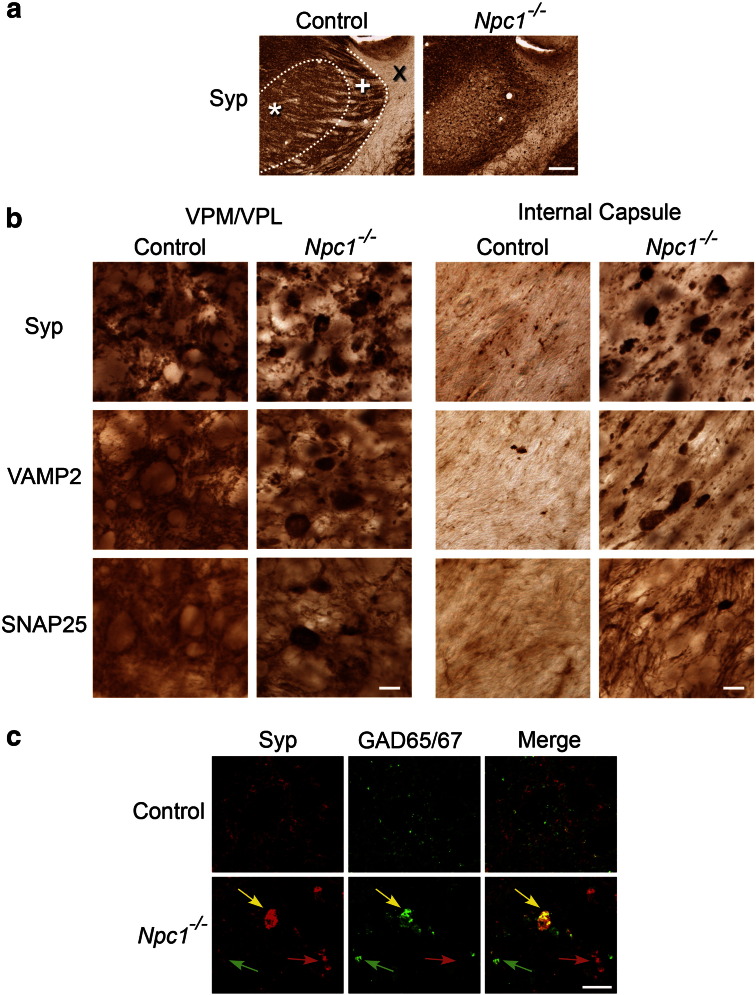
Altered distribution of presynaptic markers in *Npc1^−/−^* mice. **a** Immunohistochemical staining for synaptophysin (Syp) at the end stage of disease revealed aggregates of immunoreactivity, most prominently in the internal capsule (X), reticular (+) and ventral posterior (*) nucleus of the thalamus in *Npc1*^*−/−*^ mice. **b** Higher power pictures from the ventral posterior nucleus (VPM/VPL) and internal capsule demonstrate these large aggregates to be immunoreactive for three presynaptic markers; synaptophysin, VAMP2 and SNAP25. **c** Dual channel immunofluorescence staining for the presynaptic marker synaptophysin and the GABAergic marker GAD65/67 reveal partial co-localisation of synaptic aggregates with GABAergic axonal spheroids in the VPM (ventral medial posterior nucleus of the thalamus) of *Npc1*^*−/−*^ mice. Yellow arrows indicate aggregates immunoreactive for both the synaptic marker and GAD65/67, red arrows indicate aggregates only immunoreactive for synaptophysin/VAMP2 and green arrows indicate aggregates only immunoreactive for GAD65/67. (Scale bar in **a** = 200 μm; in **b** and **c** = 10 μm).

Our findings of white matter pathology, and existing evidence for axonal damage in *Npc1*^*−/−*^ mice (March et al., 1997; Ong et al., 2001; Takikita et al., 2004) suggests that sites distal from the cell body may be pathologically affected in these mice. To further understand any neurodegenerative process in these structures, we examined a representative selection of synaptic markers normally expressed in either pre- or post- synaptic compartments. While examination of two post-synaptic markers (Homer-1 and gephyrin) revealed a pattern of immunoreactivity in *Npc1*^*−/−*^ similar to that seen in control mice (data not shown), large clusters of three pre-synaptic markers [synaptophysin, synaptobrevin (vesicle associated membrane protein-2, VAMP2), and soluble synaptosomal-associated protein of 25 kDa (SNAP25)] were evident in both subcortical grey and white matter in *Npc1*^*−/−*^ mice. In control mice, staining for these presynaptic markers revealed punctate immunoreactivity within the neuropil, leaving neuronal soma as pale unstained structures in grey matter regions and very little staining in white matter (Fig. 9). In marked contrast, in *Npc1*^*−/−*^ mice aggregates of immunoreactivity appeared most prominently in the internal capsule and spread through the reticular (Rt) and the VPM/VPL thalamic nuclei (Figs. 9a–b). Additionally, in the VPM/VPL of these mutant mice there was a general decrease in the level of synaptophysin and VAMP2 immunoreactivity in the neuropil, different to the normal expression of these synaptic markers (Figs. 9a–b). These large aggregates were already evident in 3 week-old mice and were most frequently immunoreactive for synaptophysin and VAMP2, with fewer SNAP25 positive clusters (Fig. 9b). In *Npc1*^*−/−*^ mice, aggregates of presynaptic markers were abundant in many of the regions that displayed reactive gliosis, including the striatum, substantia nigra, white matter tracts (Fig. 9a, x internal capsule) and the thalamus (Fig. 9a, * VPM/VPL). In contrast, only very occasional aggregates were evident in the cortex and hippocampus.

**Fig. 10:**
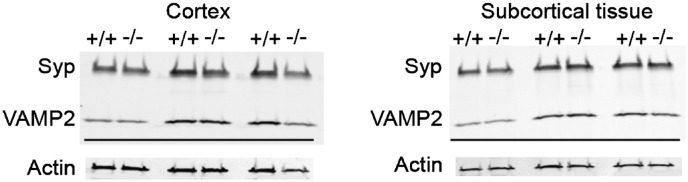
No change in the level of expression of synaptic markers. Western blots on enriched synaptic fractions from cortical and subcortical forebrain indicate no change in the level of expression of synaptic markers. Actin was used as a loading control. Syp; Synaptophysin.

To determine whether these changes were associated with an overall increase or decrease in presynaptic marker expression, enriched synaptic fractions from cortical and subcortical forebrain tissue were run on western blots and probed for synaptophysin and VAMP2 (Fig. 10). Quantitative immunofluorescence scanning of these blots revealed no overall difference in the level of these pre-synaptic markers after normalisation to actin in either the cortex or subcortical tissue. This suggests a redistribution rather than up- or down-regulation of these presynaptic markers.

The large size, morphology and distribution of the aggregates of presynaptic markers are characteristic of the axonal spheroids that develop in neurons in many neuropathic lysosomal storage disorders, including NPC (March et al., 1997; Walkley et al., 1991; Zervas et al., 2001). To examine this phenotype in more detail, we used dual channel immunofluorescence staining of GAD65/67 (as a marker of GABAergic neurons) and synaptophysin. This revealed axonal spheroids immunoreactive for both GAD65/67 and synaptophysin in the VPM/VPL (Fig. 9c yellow arrows). However, not all GABAergic spheroids contained synaptophysin (green arrows) and some clumps of synaptophysin were evident in non-GABAergic cells (red arrows).

These data suggest that the neurodegenerative process in *Npc1*^*−/−*^ mice also involves sites distal from the cell body, with pronounced aggregation of synaptic markers displayed in the regions with the most pronounced reactive gliosis.

## Discussion

In this study we have examined progressive hallmarks of neuropathology in *Npc1*^*−/−*^ mice with the aim of understanding the selective impact of loss of NPC1 upon the CNS. This has revealed that in addition to motor structures (such as the cerebellum and basal ganglia, which are characteristic of NPC), marked pathology is also evident within interconnected sensory pathways. Our survey of glial markers highlighted that thalamic nuclei relaying sensory information are an early site of pathological insult, with neuron loss already evident in the youngest mutant mice examined. In addition to identifying this regionally localised pathology, we provide evidence to suggest that neurodegenerative insults are present in the more distal parts of neurons before the cell body is affected in *Npc1*^*−/−*^ mice.

### Localised pathology within interconnecting sensory pathways

NPC disease primarily results in motor impairment associated with cerebellar degeneration (Higashi et al., 1993; Sarna et al., 2003), and neuropathology in related motor structures such as the basal ganglia (Baudry et al., 2003). Apart from these insults upon the motor system, neuron loss is also reported in the thalamus, cortex and selected brain stem nuclei at disease end stage (German et al., 2001; Luan et al., 2008; Yamada et al., 2001). However, the only report to examine the events preceding this neuron loss outside the cerebellum was Yamada et al. (2001), who showed evidence of early neuron loss in the thalamus from 4 weeks of age.

Using unbiased stereological methods, we have confirmed and extended these original observations (Yamada et al., 2001), revealing that specific populations of relay neurons are lost early in disease progression in the thalamus of *Npc1*^*−/−*^ mice (Fig. 6). Furthermore we have obtained significant new data for other neuropathological events that are most pronounced in the sensory thalamocortical system of these mice. Indeed, our data indicate that these pathways are an early pathological target of disease in *Npc1*^*−/−*^ mice; we demonstrate that there is already significant neuron loss in multiple thalamic nuclei in 3 week-old *Npc1*^*−/−*^ mice (Fig. 6) long before the onset of symptoms is apparent. Furthermore, by also examining the onset of neuron loss in the cortex, we have defined the staging of neuropathology within these interconnected thalamocortical pathways in *Npc1*^*−/−*^ mice. We demonstrate that somatosensory relay neurons are lost first in the VPM thalamic nuclei, followed by the sequential loss of feedback cortical neurons in lamina VI of the S1BF and finally loss of lamina IV granule neurons, which receive input from the VPM/VPL (Fig. 6f). These new data highlight that sensory thalamic pathways are particularly vulnerable to loss of NPC1 resulting in neurodegeneration in *Npc1^−/−^* mice.

### Prominent reactive gliosis in the thalamocortical system

Neurons are just one component of the brain, relying upon their close interactions with glia, the support cells of the brain. While each type of glial cell plays important roles within the healthy CNS, in disease states they may alter their morphology, cell surface antigen expression or in the case of microglia, proliferate (Bradl and Lassmann, 2010; Raivich et al., 1999; Seth and Koul, 2008). In *Npc1^−/−^* mice there are a number of previous reports demonstrating an upregulated immune response (Baudry et al., 2003; German et al., 2001; German et al., 2002; Luan et al., 2008; Suzuki et al., 2003), but this is the first detailed description of glial activation in relation to the neuron loss that subsequently occurs. Recent evidence suggests that this glial activation is not the primary contributor to neuron loss, since neuron-specific restoration of *Npc1* expression in transgenic mice is sufficient to mitigate their disease (Lopez et al., 2011). Likewise selective deficiency of *Npc1* in Purkinje neurons of the cerebellum results in selective Purkinje cell loss while surrounded by wild-type glial cells (Ko et al., 2005). Nevertheless, there is in vitro and in vivo evidence to suggest that the immune response does modify disease progression and pathology. Firstly, when wild-type neurons were cultured with *Npc1*^*−/−*^ astrocytes, *Npc1* deficiency in astrocytes resulted in decreased neurite growth in wild-type neurons (Chen et al., 2007). *Npc1^−/−^* astrocytes were demonstrated to release less estradiol and importantly, giving additional estradiol to neonatal *Npc1^−/−^* mice improved their clinical outcome, suggesting a pathogenic role of this estradiol deficiency. Secondly, reducing the immune response in *Npc1*^*−/−*^ mice with anti-inflammatory drugs was able to significantly prolong the life span of diseased mice (Smith et al., 2009). Although it is likely that glial activation occurs secondary to an, as yet unidentified, primary neuron-specific insult in *Npc1^−/−^* mice, its relationship to neuron loss has not been investigated in detail and our study provides a robust basis to understand the staging of these events.

Our data reveal that neuron loss in sensory thalamic nuclei is associated with the most intense immunoreactivity for astrocyte and microglial markers (Figs. 2, 3), however the relationship between these events is complex. For example at 3 weeks of age the VPM shows more intense microglial activation and astrocytosis compared to the adjacent VPL (Figs. 2, 3). Surprisingly, the onset of neuron loss was reversed in sequence in these two nuclei with reduced neuron number already evident in the VPL at 3 weeks of age, and subsequently becoming significant in the VPM at 6 weeks of age (Fig. 6). However, in the cortex, although a low level of glial activation preceded neuron loss, more intense reactive gliosis (in lamina VI of the S1BF) only became evident after the onset of neuron loss in this region of *Npc1*^*−/−*^ mice (Fig. 6). Our examination of the precise staging of events demonstrate that the relationship between glia and neurons varies between brain regions in *Npc1*^*−/−*^ mice, suggesting that the cues causing glial reactivity may differ between brain regions.

By examining pathological events at different stages of disease, our data reveal that pathological insults appear to be particularly pronounced within interconnecting pathways. For example the early and pronounced pathology in the VPM/VPL was followed by localised gliosis in lamina VI of the S1BF, where neurons provide feedback input to the thalamus. This may simply reflect the intrinsic vulnerability of these particular neuron populations, but also raises the possibility that these neuropathological changes have a trans-synaptic component. This might involve the loss of trophic influences upon afferent neurons that are already compromised by *Npc1* deficiency and the recent development of mice in which *Npc1* expression can be inducibly expressed in specific neuron populations (Lopez et al., 2011) will be a valuable to address such issues.

How this pathology of sensory thalamic pathways impacts upon the disease phenotype is unclear. In human NPC disease, imaging studies have found that the thalamus amongst other structures has an altered signal compared to controls (Huang et al., 2011; Walterfang et al., 2010). Interestingly, there is evidence for selective pathology at multiple steps of sensory pathways in *Npc1^−/−^* mice, with degeneration at the level of the sensory dorsal root ganglion (Ohara et al., 2004) and brain stem nuclei (Luan et al., 2008). Our data shows that the somatosensory thalamocortical pathway (VPM/VPL connecting to S1BF) displayed the most pronounced pathology, with the interconnected Po nucleus that also relays somatosensory input similarly displaying localised reactive gliosis (Table 1). The somatosensory system processes proprioceptive, nociceptive and tactile information and in rodents the S1BF is involved with processing sensory input from the whiskers (O'Leary et al., 1994). Although sensory abnormalities are not commonly reported in NPC disease, if such defects do occur, they are likely to go unrecognised or be masked by severe neurological deterioration. Considering the early and profound effects upon somatosensory pathways in *Npc1^−/−^* mice, it would be informative to examine whether subtle pain or somatosensory abnormalities, are also evident in these mice.

### Localised rod microglia, a distinct immune reaction?

In addition, our data also reveal new information about the nature of this immune response in *Npc1^−/−^* mice. Our use of F4/80 and CD68 immunohistochemistry revealed microglia with an abnormal morphology in the striatum and cortex of *Npc1*^*−/−*^ mice, with long, straight rod-like processes forming into clumps (Figs. 5, 7). Rod microglia are reported to occur in models of ischaemic stroke (Ng and Ling, 2001; Zhang et al., 1997) and extend processes along degenerating fibre tracts (Schroeter et al., 2002) or along blood vessels (Lin et al., 1998). The rod microglia observed in *Npc1*^*−/−*^ mice were very large, extending beyond the size of multiple amoeboid microglia and therefore appear much larger than those reported in stroke (Schroeter et al., 2002; Zhang et al., 1997). This novel observation raises the possibility that these microglia are surrounding degenerating axons (Baudry et al., 2003; Ong et al., 2001) and raises the question of why they are apparent in such a localised fashion in the ventral lateral striatum.

### Reactive glia in the basal ganglia of *Npc1*^*−/−*^ mice

In addition to the rod-like microglia in the striatum, we also found localised activation of glia within other structures of the basal ganglia of *Npc1*^*−/−*^ mice including the globus pallidus and the substantia nigra (Fig. 7). Astrocytosis and microglial activation have previously been reported in these brain regions (Baudry et al., 2003; Luan et al., 2008) and this was demonstrated to be associated with neuron loss in the substantia nigra (Baudry et al., 2003; Luan et al., 2008; Ong et al., 2001). The basal ganglia are associated with the modulation of motor control (DeLong and Wichmann, 2009; Mink, 1996) and signs of basal ganglia dysfunction, including dystonia are a common clinical feature of NPC (Imrie et al., 2007). This localised basal ganglia pathology is likely to be associated with broader pathology within these interconnected motor pathways, with the cerebellum known to be severely affected in NPC (Higashi et al., 1993; Sarna et al., 2003) and localised reactive gliosis within thalamic nuclei relaying motor information (Ventral lateral nucleus; Table 1). Interestingly, our data have revealed that the dorsal part of the substantia nigra showed the greatest intensity of CD68 and GFAP immunoreactivity and similarly, the most marked microglial activation in the striatum was localised to the ventral lateral part (Fig. 7). As well as being associated with motor functions, the basal ganglia have more recently been demonstrated to be involved in behaviours such as motivation, emotional drive, planning and cognition (Graybiel, 2005; Haber and Calzavara, 2009). Our data suggest that pathological processes impact upon highly localised pathways within the basal ganglia. Dysfunction of these specific pathways could potentially contribute to cognitive and behavioural symptoms, in addition to the motor symptoms evident in NPC disease (Garver et al., 2007; Imrie et al., 2007).

### Synaptic abnormalities occur early in disease progression

While our counts of neuron numbers have provided a sensitive measure of neuron loss (Fig. 6), degeneration of the neuron soma is the terminal step in the neuropathological cascade. In *Npc1*^*−/−*^ mice silver staining reveals degenerating neuron terminals (Ong et al., 2001), and demyelination has been reported early in disease progression (German et al., 2001; Takikita et al., 2004; Weintraub et al., 1987) suggesting that the degenerative process may originate in the distal parts of the neuron. Consistent with these data, our study revealed atrophy of white matter structures early in disease progression (Figs. 8c–d) and a particularly marked astrocytosis and microglial activation in the white matter, with microglia already fully transformed into amoeboid microglia by 3 weeks of age (Fig. 8a). Likewise in the human disease, a widespread white matter atrophy has been found in patients (Walterfang et al., 2010).

With regard to the synapse, the NPC1 protein is present in terminal axons and synapses (Karten et al., 2006; Patel et al., 1999; Xu et al., 2010) and presynaptic terminals are enlarged and show signs of degeneration (Ohara et al., 2004). Importantly, these changes appear to be functionally relevant, with *Npc1* deficient synapses showing increased spontaneous activity (Wasser et al., 2007) and delayed exocytosis (Xu et al., 2010). Cholesterol is important in the correct functioning of synapses and cholesterol depletion in nerve terminals due to the defect in cholesterol trafficking in *Npc1*^*−/−*^ brain is thought to be responsible for this synaptic dysfunction (Wasser et al., 2007). Our survey of pre- and post-synaptic proteins has demonstrated a marked redistribution of presynaptic proteins that occurs early in disease progression in *Npc1*^*−/−*^ mice (Fig. 9). Our novel data details that distinct synaptic phenotypes are evident within different regions of the CNS; presynaptic protein redistribution was profound early in disease in subcortical grey and white matter structures (Fig. 9), whereas the cortex remained relatively spared, even at disease end stage. The challenge will now lie in determining if there is a mechanistic link between these events and the subsequent loss of neurons in these pathways.

Our data imply that in *Npc1*^*−/−*^ mice, a proportion of presynaptic markers may fail to reach the nerve terminals because they become aggregated within axonal spheroids in the white matter (Fig. 9c). The occurrence of axonal spheroids correlates with neurological symptoms and severity in LSD models, and therefore axonal spheroid formation is thought to contribute to neuronal dysfunction (Walkley et al., 1991). Our observation that presynaptic markers are aggregated within axonal spheroids suggests that axonal transport of these proteins is disturbed in both *Npc1*^*−/−*^ mice. Disrupted axonal transport has already been reported in *Npc1*^*−/−*^ mice (Neufeld et al., 1999; Zhang et al., 2001) and our data imply that this also results in a proportion of presynaptic proteins failing to reach their synaptic target. Furthermore a loss of presynaptic input onto neuronal soma in the thalamus may have a secondary contribution to neuron loss. Despite the marked presynaptic phenotype in *Npc1*^*−/−*^ mice, we found little evidence for postsynaptic rearrangements. This is consistent with Xu et al. (2010) who reported no difference in the expression of postsynaptic density protein PSD-95 or multiple neurotransmitter receptors in *Npc1*^*−/−*^ neurons. Their data and ours suggest that the disease process impacts the synapse in an asymmetrical fashion.

These observations of axonal and synaptic abnormalities in the thalamocortical system of *Npc1^−/−^* mice again highlight that a series of pathological events occur within these interconnected pathways. Although we have systematically mapped the order of events through these pathways, key questions remain about the apparent vulnerability of these neurons and how it relates to the catabolic defect in this disorder. For example, why do these particular neurons succumb to the disease process, while others remain unaffected, despite the same genetic deficiency being present in all cells? The simplest explanation could be that the burden of storage material could gradually increase over time until a certain threshold is reached by which individual neurons and pathways succumb to the disease. However, with the precise function of NPC1 remaining elusive, it remains unclear whether the primary pathology is a direct result of storage material accumulation or another as yet unidentified consequence of *Npc1* deficiency. Our data provide a new perspective, revealing a number of events that occur prior to and alongside neuron loss and highlighting that these occur in a pathway dependent manner.

## Conclusion

In conclusion, this study has revealed a series of novel neuropathological alterations in the CNS of *Npc1*^*−/−*^ mice that were particularly pronounced within the sensory thalamocortical system. Our data add significantly to the current literature by demonstrating an early activation of astrocytes and microglia in *Npc1*^*−/−*^ mice, implicating the thalamus and basal ganglia as specific foci of disease pathology. These findings suggest that there are multiple components of this immune response, with an early low level glial activation followed by the sequential appearance of localised patches of intense glial reactivity becoming evident with subsequent disease progression. We demonstrate that these regions, which display particularly pronounced reactive gliosis also display rearrangement of presynaptic markers, suggesting a pathological impact upon distal parts of the neuron. Examination of the onset and progression of events through the disease course suggests that pathology progresses through the interconnected nuclei of the thalamocortical system. Why these interconnected pathways are vulnerable to disease remains unclear. Further understanding of the impact of loss of *Npc1* upon the brain will help to elucidate the degenerative process in NPC and is necessary to understand and why loss of this gene has such severe neurodegenerative consequences.

## Figures and Tables

**Table 1:** Tabular representation of the distribution of localised astrocytosis and microglial activation in thalamic nuclei in *Npc1*^*−/−*^ mice. A scoring of the intensity of immunoreactivity for GFAP (astrocytes), F4/80 and CD68 (microglia) in different thalamic nuclei is shown at the 3 age points examined. The number of + signs denotes the intensity of GFAP, F4/80 and CD68 + ve staining along a scale as follows: +++++, nucleus full of densely packed and intensely stained + ve cells; +++, + ve cells covering at least half the nucleus; blank, no or very few positive cells detected.

Grouping	Modality	Thalamic nuclei	Intensity of gliosis		
Early: 3 wks	Mid: 6 wks	End: 9 wks
Sensory	Somatosensory	Ventral lateral posterior (VPL)	++	++++	+++++
Somatosensory	Ventral medial posterior (VPM)	++	++++	+++++
Somatosensory	Posterior thalamic nuclear group (Po)	+	++	+++
Auditory	Medial geniculate (MGN)	++	+++	++++
Visual	Dorsal lateral geniculate (LGNd)	+	++++	++++
Visual	Ventral lateral geniculate (LGNv)		+	+
Effector	Motor	Ventral lateral (VL)	++	+++	+++
Limbic	Drive, motivation, emotion	Medial dorsal central (MDC)	+	++	++
Physiological response to stress	Anteroventral (AV)		+	+
Visual and Limbic connections	Lateral dorsal (LP)	+	++	++
Anteromedial			
Associative	Somatosenory and visual integration	Lateral posterior (LP)	+	++	++
Olfaction	Submedial (Sub)		+	++
	Medial dorsal lateral, medial (MDL, MDM)			
Reticular	Arousal, rhythmicity	Reticular (Rt)	+	++++	++++
Intralaminar	Arousal, attention	Parafasicular (Pf)			+
Intralaminar	Autonomic drive	Paracentral (PC)			
Effector	Motor	Ventromedial (VM)			
